# Looseness Identification of Track Fasteners Based on Ultra-Weak FBG Sensing Technology and Convolutional Autoencoder Network

**DOI:** 10.3390/s22155653

**Published:** 2022-07-28

**Authors:** Sheng Li, Liang Jin, Jinpeng Jiang, Honghai Wang, Qiuming Nan, Lizhi Sun

**Affiliations:** 1 National Engineering Research Center of Fiber Optic Sensing Technology and Networks, Wuhan University of Technology, Wuhan 430070, China; lisheng@whut.edu.cn (S.L.); jiangjp2812@whut.edu.cn (J.J.); wanghh@whut.edu.cn (H.W.); 2 School of Information Engineering, Wuhan University of Technology, Wuhan 430070, China; jl1290787604@whut.edu.cn; 3 Department of Civil and Environmental Engineering, University of California, Irvine, CA 92697-2175, USA; lsun@uci.edu

**Keywords:** feature identification, track fastener looseness, distributed vibration, convolutional autoencoder network, pseudo-Hilbert scan, ultra-weak fiber optic Bragg grating

## Abstract

Changes in the geological environment and track wear, and deterioration of train bogies may lead to the looseness of subway fasteners. Identifying loose fasteners randomly distributed along the subway line is of great significance to avoid train derailment. This paper presents a convolutional autoencoder (CAE) network-based method for identifying fastener loosening features from the distributed vibration responses of track beds detected by an ultra-weak fiber Bragg grating sensing array. For an actual subway tunnel monitoring system, a field experiment used to collect the samples of fastener looseness was designed and implemented, where a crowbar was used to loosen or tighten three pairs of fasteners symmetrical on both sides of the track within the common track bed area and the moving load of a rail inspection vehicle was employed to generate 12 groups of distributed vibration signals of the track bed. The original vibration signals obtained from the on-site test were converted into two-dimensional images through the pseudo-Hilbert scan to facilitate the proposed two-stage CAE network with acceptable capabilities in feature extraction and recognition. The performance of the proposed methodology was quantified by accuracy, precision, recall, and F1-score, and displayed intuitively by t-distributed stochastic neighbor embedding (t-SNE). The raster scan and the Hilbert scan were selected to compare with the pseudo-Hilbert scan under a similar CAE network architecture. The identification performance results represented by the four quantification indicators (accuracy, precision, recall, and F1-score) based on the scan strategy in this paper were at least 23.8%, 9.5%, 20.0%, and 21.1% higher than those of the two common scan methods. As well as that, the clustering visualization by t-SNE further verified that the proposed approach had a stronger ability in distinguishing the feature of fastener looseness.

## 1. Introduction

The characteristics of small space, long mileage, and difficulty in personnel evacuation determine that it is more challenging to safeguard the underground structure of the subway than the ground building. To ensure subway safety, a wide range of research efforts has been undertaken in the fields of subway fires [1,2,3], structural safety [4,5,6,7], and illegal invasion [8,9,10,11,12]. Among the objects of concern in the field of structural safety monitoring, the track used to guide the train and bear the moving loads transmitted by the wheels plays a vital role in the operation of the subway. In the absence of extensive and meticulous inspection and maintenance, the long-term repeated impact of subway trains will undoubtedly reduce the strength and stability of the track structure. In severe cases, the vibration of the track system caused by the interaction between the train and the track will cause the rail fasteners to loosen, rupture, and even lead to catastrophic derailment accidents [13]. In related reports on train derailment, the research status and major problems were reviewed in [14]. For the detection method aiming for preventative maintenance, the traditional inspection regime is usually labor-intensive and can be significantly expensive for the rail operator [15]. In addition to manual-based periodic inspection methods, Ikshwaku et al. [16] reported the feasibility of using drones to monitor railway-related infrastructure, which is obviously not suitable for monitoring subway lines in tunnels. Moreover, the image-based detection techniques were compared in [17], which are susceptible to the interior environment of the subway tunnel.

Since the tracks are fastened to the track bed by fasteners, it is possible to obtain the fastener condition by monitoring the structural vibration response of the track bed caused by passing trains in terms of the relationship between the vibration response and the structural state. According to the reports in [11,12,18], the ultra-weak fiber optic Bragg grating (FBG) sensing technology [19] is a feasible way to collect the distributed vibration of the track bed along the subway line. To avoid the vibration characteristics caused by fastener loosening being submerged in the vibration response of the track bed, feature extraction should be the most intuitive idea that can improve the effectiveness and efficiency of the fastener’s looseness identification. Numerous reports [20,21,22,23] have demonstrated the good performance of the convolutional neural network (CNN) in various application domains. For the CNN-based feature extraction of the time-series signal, representative research on feature extraction directly on one-dimensional signals can be found in [24,25,26]. Moreover, there are reports [27,28,29] on feature extraction based on two-dimensional images converted from one-dimensional signals. However, CNN-based methods require supervised learning based on a large number of labeled samples, which is not suitable for the situation that relies heavily on sufficient fastener looseness samples. Also, the above feature extraction methods are difficult to fully grasp or retain the spatial information between sampling points in the original one-dimensional signal. In contrast, the convolutional autoencoder (CAE) network [30] has low dependence on labeled samples for training; that is, CAE networks only need a small number of labeled samples for classification research on the dataset of interest [31,32]. Additionally, despite the lack of practical engineering verification cases concerning the signal dimensional conversion, it has been reported that the encoding operation of a one-dimensional signal based on a pseudo-Hilbert scan can theoretically preserve more of the sample’s original feature [33,34,35]. This means that the encoding results of the two-dimensional images are beneficial to the signal feature extraction based on the CAE network.

Therefore, the purpose of this paper is to propose a method for identifying the looseness of subway fasteners based on the CAE network combined with the pseudo-Hilbert scan for the distributed vibration response detected by the ultra-weak FBG sensing array. That is, the original one-dimensional samples are converted into two-dimensional images through the pseudo-Hilbert scan, and then the converted results are used as inputs of the CAE network to perform the study of fastener looseness identification. The design and arrangement of the field experiment used to generate the fastener looseness dataset detected by the ultra-weak FBG sensing array make up the second part of this paper, followed by methodology details for identifying the looseness of subway fasteners, including the pseudo-Hilbert scan operation and the descriptions of CAE network architecture. Finally, the effectiveness and performance superiority of the proposed method are primarily quantified by accuracy, precision, recall, and F1-score, and the ability to distinguish target signals is further visualized by t-distributed stochastic neighbor embedding (t-SNE) [36].

## 2. Field Experiment for Generating the Fastener Looseness Dataset

### 2.1. Background of Vibration Signal Acquisition

**Figure 1:**
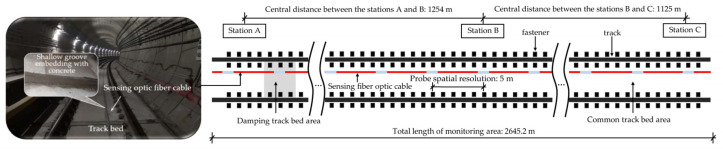
Field deployment of the ultra-weak FBG sensing array for acquiring vibration responses of track beds.

The vibration signals used in this study were derived from the monitoring data of an actual subway tunnel structure. Before the operation of the subway, an ultra-weak FBG sensing optic fiber with armored protection using a layer-stranding structure with a loose tube was fixed along the track bed surface of the selected tunnel segments. As shown in Figure 1, the monitoring area covers three underground stations of the subway line, with a total length of nearly three kilometers. The shallow groove embedding was adopted to affix the sensing array to secure a better vibration response of the track bed. The effect of this fixation method was discussed in [12]. The previous study [37] revealed the repeatability of such a sensor is around 3.41 nε. According to the 5 meters spatial resolution of the probes in the sensing optic fiber, more than 500 consecutive regions monitored along the track bed can feedback the structural vibration response based on the interrogated address of the light interference [19]. When a train passed, the structural vibration response triggered in each monitoring area was acquired at a 1 kHz sampling rate. The collected data was transmitted to the remote monitoring center for processing by the demodulator and server.

### 2.2. Design and Arrangement of Experimental Cases

At present, the subway line has already been in operation. To secure the safety of train operations on the following day, various inspections are usually conducted during the subway outage in the early hours of the morning. Therefore, the field experiment for collecting the dataset of fastener looseness was performed in this inspection window. As shown in Table 1, the distributed vibration responses of the track bed corresponding to three states were collected in total in the field test.

**Figure 2:**
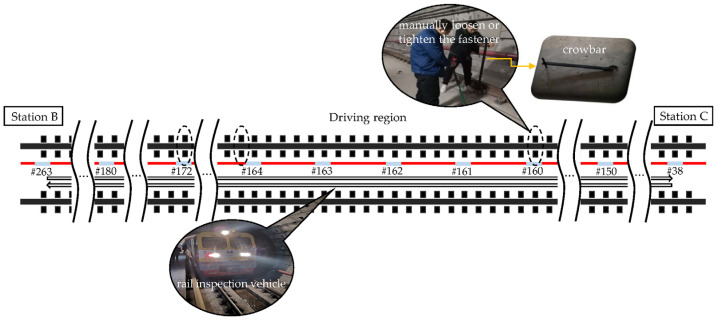
Position of the selected fasteners and detection area of the ultra-weak FBG sensing array.

The three locations within monitoring areas #160, #164, and #172 shown in Figure 2 were randomly selected to perform fastener looseness. The selected sites are all located in the common track bed area, which accounts for the majority of the subway lines. The rail inspection vehicle with a single carriage of about 5 meters in length was utilized to excite the vibration responses of the track bed under the three test states illustrated in Table 1. In each location, a crowbar was employed to manually loosen or tighten a pair of fasteners symmetrical on both sides of the track. To collect more samples within the limited experimental period, for fastener states 2 and 3 in Table 1, the 6 fasteners in the three locations were loosened or tightened together. Moreover, in areas covering the fasteners of interest, the rail inspection vehicle passed twice in two driving directions at a speed below 25 km/h, respectively. To ensure the quality of the collected signals, the vehicle stopped for 3 min each time it arrived at the station and then traveled in the direction specified in Table 1.

### 2.3. Dataset and Its Division and Usage

According to the field records in Table 1, 12 groups of distributed vibration signals of the track bed structure caused by the passage of the rail inspection vehicle were collected. The vibration responses of the track bed within the range of #150 to #180 in the monitoring areas were selected to establish the dataset for the subsequent study. Among them, the signals in regions #160, #164, and #172 corresponding to fastener state 2 in Table 1 were considered to have fastener looseness characteristics. The responses under fastener states 1 and 3 for regions #160, #164, and #172, along with the vibration responses from the other regions, together constituted the samples of fasteners in the normal state. 

**Figure 3:**
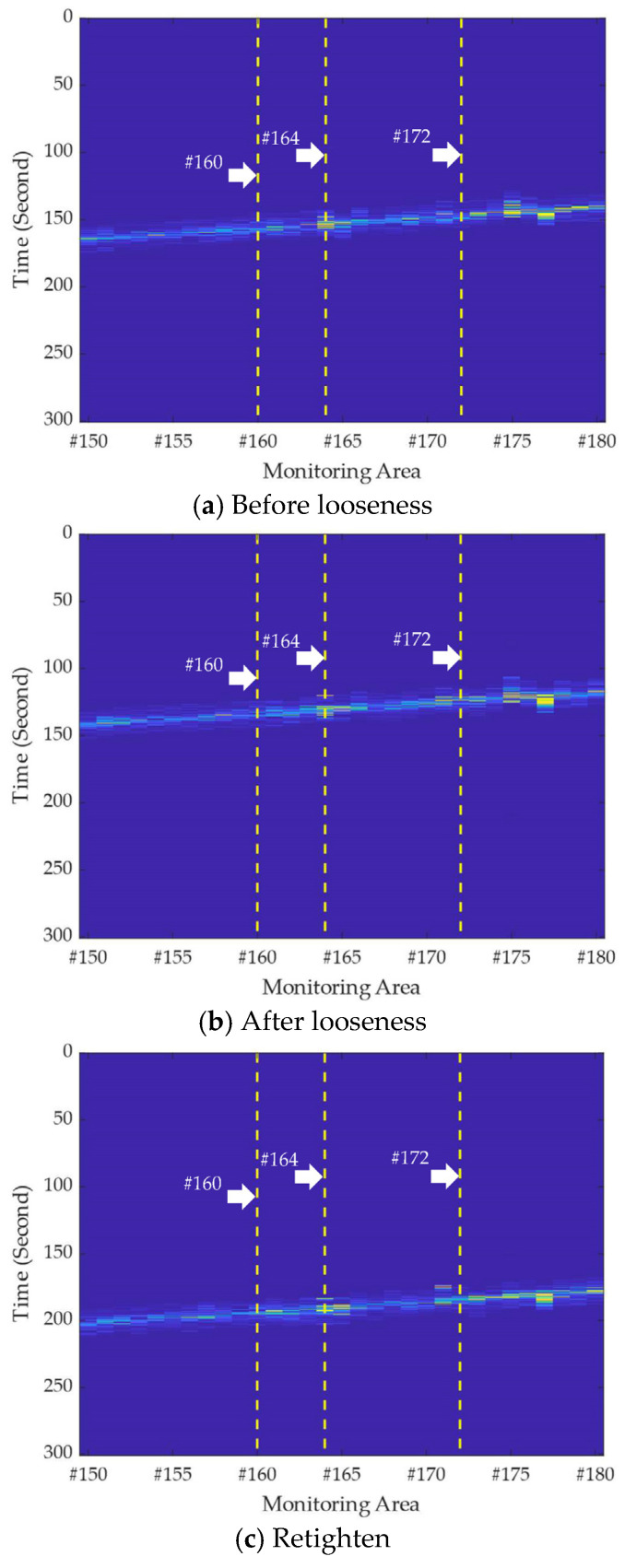
Vibration intensity versus space and time under the moving of rail inspection vehicle corresponding to (**a**–**c**) three fastener states.

The raw output result for each record in Table 1 can be plotted as a waterfall diagram of the vibration response versus time and space under the vehicle moving loads between stations B and C. Based on Table 1, Figure 3 depicts the waterfall diagrams of monitoring areas from #150 to #180 corresponding to the three fastener states. Here, the driving records for illustrating the three fastener states in Figure 3 were randomly selected from Table 1 under the premise of maintaining a consistent driving direction. Unfortunately, from the subplots of Figure 3, it is difficult to distinguish the negative influence of loose fasteners on the distributed vibration of the track bed.

**Figure 4:**
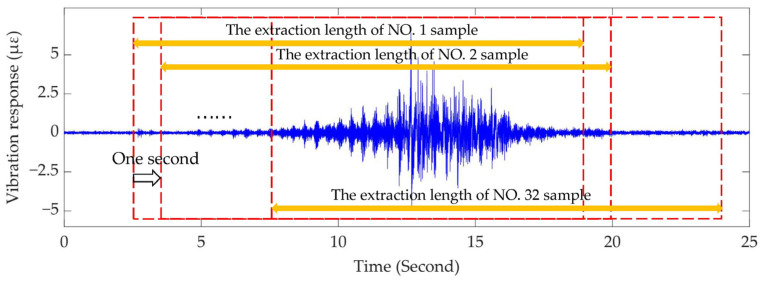
Data augmentation strategy for monitoring area #160 under fastener loose state.

Since there were only 12 original samples of the loose state of the fasteners, data augmentation was performed on the vibration signals of the monitoring areas corresponding to the three fastener looseness positions. Specifically, taking the original vibration response stimulated by the vehicle in monitoring area #160 as an example shown in Figure 4, the enhanced signals were generated by window-by-window translation for the collected signal, and the shift interval of the translation window was set to a one second step. Additionally, the width of the window ensures that the main vibration characteristics caused by the passage of the rail inspection vehicle can be preserved. Based on this strategy, the vibration response concerning the monitoring area corresponding to each fastener looseness position was represented by 32 samples in this paper.

The dataset of the normal state of the fastener came from two parts: the responses of the three monitoring areas #160, #164, and #172 under fastener states 1 and 3, and the responses of the remaining 31 monitoring areas under three specified states. Thus, the composition and size of the experimental dataset were given in Table 1. Here, to meet the consistency requirements of the CAE network for the dimension of the input sample, all samples described in Table 2 are kept at the same length with 20,164 sampling points.

To perform the subsequent study, the dataset described in Table 2 was divided into two parts to perform training and testing based on the commonly used ratio [38] of 7:3. As given in Table 3, the training set was used to perform the two-stage CAE network training. During the pre-training stage, all the training samples participate in the automatic extraction of hidden features of signals through unsupervised learning. Then, a fine-tuning of the network performance was performed in the second training stage of the CAE network. Here, to reduce the influence of asymmetry between sample sizes on fine-tuning results, the data balance between the two labels was considered. That is, 67 samples were randomly selected from the training dataset labeled B, together with all the training datasets labeled A, to form the labeled dataset for fine-tuning the network based on supervised learning. Moreover, to reduce the sensitivity of network performance to data partitioning and to obtain as much valid information as possible from the enhanced data, ten-fold cross-validation was used in this study. Additionally, a type of min-max normalization [39] was adopted to normalize all the sample amplitudes to the range of 0–1 to increase the learning efficiency of the proposed CAE network.

## 3. Methodology for Identification of Fastener Looseness

### 3.1. Pseudo-Hilbert Scan Operation

The pseudo-Hilbert scan can encode the original vibration response of the track bed by establishing a space-filling curve that matches the length of the one-dimensional sample sequence, namely, the length *L* and width *W* of the space-filling curve should meet Equation (1):

(1) L×W≥N

where the result of L×W is the minimum value that meets Equation (1), *N* represents the sequence length of the original sample, and *L*, *W*, and *N* should all be integers.

**Figure 5:**
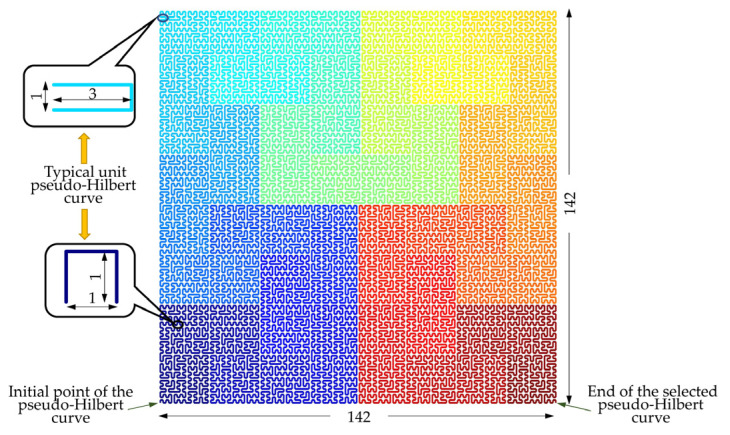
The pseudo-Hilbert curve for encoding the one-dimensional vibration response.

According to the description in Section 2.3, the sequence length *N* of each original sample was set to 20,164. Therefore, the dimension shape of the space-filling curve can be set to 142 × 142. Under this condition, the result of L×W was equal to *N*, which satisfied the instruction aforementioned. As well as that, the study in [40] revealed that the space-filling curve with equal length and width parameters ensured a better space clustering effect between the original sample points evaluated by mean square Euclidean distance [41]. In the implementation process, a null matrix of dimension 142 × 142 for the pseudo-Hilbert scan was created. Then, the points within the null matrix that were used to form the initial space-filling curve were connected. According to the pseudo-Hilbert curve decomposition rule, the initial space-filling curve was gradually decomposed until obtaining the unit pseudo-Hilbert curve with a length or width of 1 or 2 [42]. Based on the Hilbert flipping operation [40], each basic unit pseudo-Hilbert curve was connected sequentially to form the pseudo-Hilbert curve as shown in Figure 5 for encoding the one-dimensional signal samples. The signal-to-image conversion operation based on the curve direction shown in Figure 5 can generate the input expression of the subsequent CAE network, where the evolution processes of the step-by-step decomposition and the sub-region connection are represented by different color blocks.

### 3.2. Establishment of CAE Network

**Figure 6:**
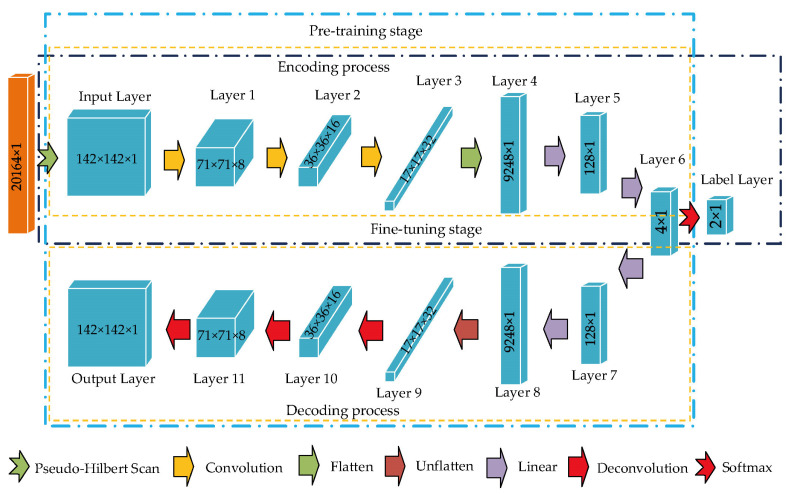
The proposed two-stage training CAE network architecture.

Based on the report in [30,43] and the current experimental hardware environment (Dell PowerEdge T630 server) that was composed of a graphics processing unit (GPU) core (GTX 1080 Ti) with twelve 2.20 GHz processors (Intel Xeon E5-2650 v4), as shown in Figure 6, the proposed CAE network consisting of two training stages was established. Both for the pre-training and fine-tuning stages, the converted samples with the dimension of 142 × 142 × 1 processed by the pseudo-Hilbert scan were put into the input layer. In the pre-training stage, the established network used 2 convolution layers, 1 flatten layer, 3 linear layers, 1 unflatten layer, and 2 deconvolution layers in sequence to implement the encoding and decoding process for input samples. The goal of network training in this stage was to ensure that the prediction error exhibited a rather weak fluctuation, that is, to ensure that the difference between the predicted sequence and the input normal sample was small. During the feature extraction process based on unsupervised learning, the training dataset defined in Table 3 was used to preliminarily search the hyperparameters of the proposed CAE network. To suppress the occurrence of overfitting, rectified linear units [44] were used as an activation function and were added after each convolutional layer. Here, the number of neurons in each layer was derived through trial and error, assisted by grid searching [45]. In the fine-tuning stage, based on the balance training dataset defined in Table 3, the softmax layer and Label layer were added at the end of the encoding process in the pre-training network to perform hyperparameter fine-tuning based on supervised learning. To meet the training objective, adaptive moment estimation [46] was selected as the optimization algorithm of the network.

## 4. Results, Analysis, and Discussion

**Figure 7:**
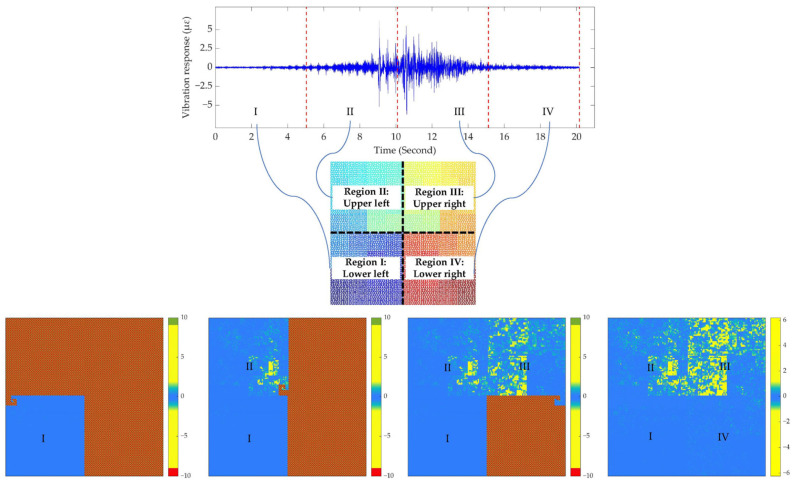
Pseudo-Hilbert scan results of a typical subway track bed vibration signal.

Based on the pseudo-Hilbert scanning operation, the responses of the sampling points of the vibration signal were sequentially placed along the curve direction depicted in Figure 5. Then, the two-dimensional image with a dimension of 142 × 142 serving as the input of the CAE network can be obtained. For the convenience of explanation, since the direction of the filling curve in Figure 5 starts from the lower left, then passes through the upper left and right regions, and finally ends at the lower right, the conversion process of signal-to-image can be illustrated by the evolution of four images in the bottom part of Figure 7. Specifically, the original vibration response of the track bed excited by the vehicle in each monitoring area can be viewed as consisting of four equal-length parts. Each one-dimensional signal sequence with a length of 5041 was converted to a two-dimensional image with a dimension of 71 × 71. It can be observed that the signals in regions II and III are more pronounced after the operation, while the conversion results in regions I and IV indicated by warm colors are not conspicuous, which conforms to the response amplitude distribution of the original signal. The image on the right side of the bottom part of Figure 7 was the final conversion result, which was used as the input of the CAE network.

According to the design in Table 3 and Figure 6, network training and testing were performed sequentially based on encoded samples under ten-fold cross-validation. With the current hardware configuration, the training runtime for the proposed network in each fold was approximately 3 h 58 min. The computation time was mainly consumed in the training phase, and the recognition prediction took only 2.43 s. For the test dataset defined in Table 3, the performance of the established CAE network was assessed by accuracy, precision, recall, and F1-score that have been widely used in the field of machine learning [47,48], and the corresponding indicator results were the average of ten-fold cross-validation and given in Table 4. From the results in Table 4, the overall performance of the network in distinguishing fastener looseness behaves well. However, directly adopting metric-based assessment is sometimes not the most appropriate choice for intuitively understanding or demonstrating network performance.

**Figure 8:**
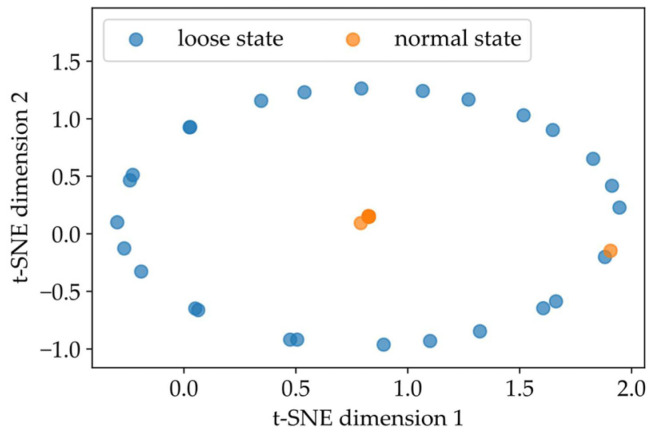
Clustering visualization results of fastener state feature based on t-SNE.

**Figure 9:**
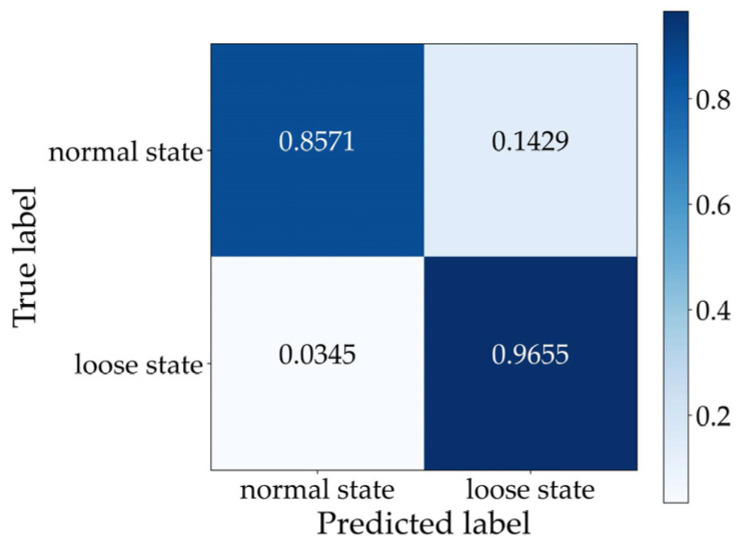
Confusion matrix of the proposed CAE network in the case of the test dataset.

To visualize the classification advantage of the CAE network proposed in this paper, the final features compressed in Layer 6 in Figure 6 were first extracted and then converted into the clustering result of a two-dimensional plane based on the t-SNE technique [49]. If the clustering effect is good, it can be considered that the established training network has a strong ability to distinguish different states of fasteners. Under ten-fold cross-validation, the typical clustering results of the vibration signals of the track bed representing the two fastener states are shown in Figure 8. Here, the worst clustering effect in the ten-repetition test was selected as the typical result. It can be observed that fastener states were clustered into two categories. Specifically, the samples reflecting loose fasteners were distributed in an ellipse topology. Moreover, it can be inferred that at least 87.39% of the samples representing the normal state of fasteners were concentrated in the center of the ellipse. Although the effect was presented in different ways, the proportion of clusters from the visualization result based on the dimensionality-reduction algorithm agreed well with the confusion matrix conclusion shown in Figure 9, which verified that the proposed CAE network was able to identify the loose state of subway fasteners.

**Figure 10:**
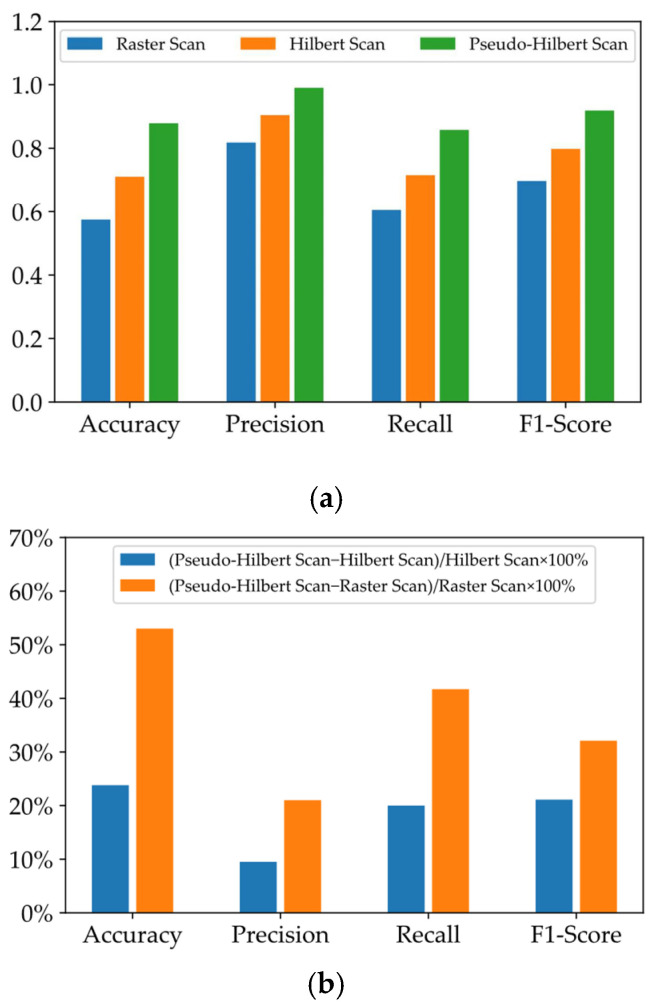
Results of (**a**) four indicators for evaluating network performance under different scanning methods and (**b**) performance superiority of pseudo-Hilbert scan.

**Figure 11:**
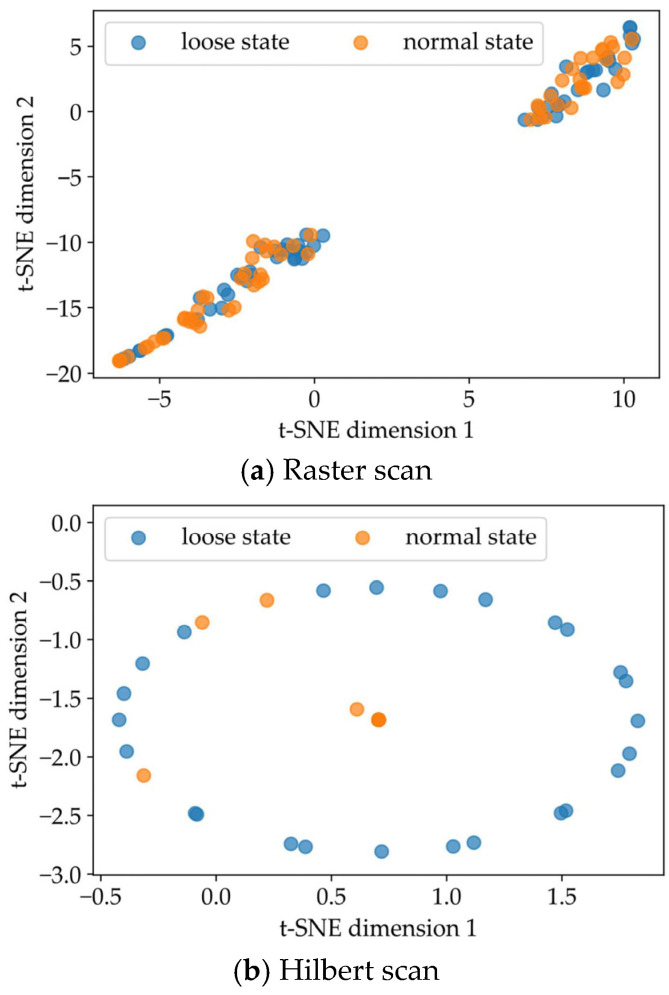
Clustering visualization results of fastener state feature of (**a**) Raster scan and (**b**) Hilbert scan based on t-SNE.

In addition, the raster scan and the Hilbert scan, two commonly used methods that can convert one-dimensional signals into two-dimensional images [50], were selected to compare with the results of the pseudo-Hilbert scan used in this paper. For the training and test samples derived from the raster scan, the same network structure in Figure 6 was used to carry out the performance evaluation. Due to the specific coding rule of the Hilbert scan [51], the vibration samples with the original sequence length of 20,164 were encoded into the shape of 256 × 256. That is, during training and testing, the network layer structure used for the Hilbert scan was similar to Figure 6 except that the dimensions of the input layer, output layer, and partial intermediate layers for matching encoding and decoding were modified. Figure 10a depicts the comparison results after ten-fold cross-validation using the same four indicators from Table 4. It can be seen that the result of the pseudo-Hilbert scan outperformed those of the other two comparison methods, and the results of the raster scan were the least ideal. Based on the similar CAE network structure, the four indicators (accuracy, precision, recall, and F1-score) as shown in Figure 10b used for performance evaluation based on the pseudo-Hilbert scan were at least 23.8%, 9.5%, 20.0%, and 21.1% higher than those of the comparison methods, respectively. Moreover, the typical visualization results based on t-SNE are shown in Figure 11. The reason for the poor effect of the raster scan can be revealed in Figure 11a. Although two clusters were formed, it was obvious that each cluster area was mixed with different fastener states, which means the clustering result is chaotic. The typical topology result based on the Hilbert scan shown in Figure 11b derived from the best clustering effect of cross-validation and behaved similarly to that in Figure 8. However, only 75.63% of the samples representing the normal state of the fastener were concentrated in the center of the ellipse, which was lower than the minimum 87.39% ratio shown in Figure 8. Thus, the results reflected in Figure 11 also demonstrated that the proposed method had a better ability to identify the fastener looseness.

Based on the above results and analysis, Table 5 gives the performance comparison between the proposed CAE network combining pseudo-Hilbert scan and the similar network combining the other two scan techniques in terms of four machine learning indicators and t-SNE-based cluster ability. From the quantitative results, the method performance proposed in this paper is significantly better than that of conventional techniques.

## 5. Conclusions

This paper reports a method aimed at identifying the subway fastener looseness based on the experimental dataset of the track bed vibration detected by the ultra-weak FBG sensing array. To the best of our knowledge, this is the first study into the looseness identification of the track fastener through the CAE network combined with the pseudo-Hilbert scan, in which the limited but precious dataset came from practical engineering. The performance indicators of the proposed methodology in terms of accuracy, precision, recall, and F1-score were at least 23.8%, 9.5%, 20.0%, and 21.1% higher than those of the traditional techniques involved in the comparison. The visualization results from t-SNE also demonstrated that the method adopted in this paper had a strong ability to extract fastener state features and led the comparison techniques by at least 11.76%. Thus, it is believed that the current study will motivate future exploration into the detection of other undesirable track states through the distributed vibration response of the track bed. However, some limitations are worth noting. Although the recognition effect has been verified experimentally, it needs to be stressed that the approved area and duration for the organized field test in this paper were constrained by the regulations of the subway operation management. Therefore, the established two-stage CAE network architecture in the proposed method still deserves to be further improved by more unknown types and degrees of fastener looseness events. When facing datasets with more noise and establishing more complex deep learning networks, data preprocessing and hyperparameter optimization based on the Bayesian method [52,53,54] may be a viable alternative. Moreover, to prevent the risk of sudden intrusion into the subway line due to the excitation of vehicle moving load when the fasteners were in a semi-loose state, this paper only discussed the identification of the complete looseness state of the fasteners. That is, the selected fasteners were moved away from the subway line immediately after being manually loosened with the crowbar. Thus, the effectiveness based on the strategy proposed in this paper for identifying the fastener in a semi-loose state deserves further attention. For the above concerns, it is necessary to further study the experiment scheme suitable for collecting more types of samples of track distribution vibration in future work, which may be helpful for the study in the areas of identification of the track bed damage and train wheel fault.

## Figures and Tables

**Table 1:** Test time record in situ under three fastener states.

State Number	Fastener State	Driving Direction	Travel Time
1	Before looseness	Station B→Station C	0:58 a.m.–1:01 a.m.
		Station C→Station B	1:04 a.m.–1:07 a.m.
		Station B→Station C	1:10 a.m.–1:13 a.m.
		Station C→Station B	1:16 a.m.–1:19 a.m.
2	After looseness	Station B→Station C	1:34 a.m.–1:37 a.m.
		Station C→Station B	1:40 a.m.–1:43 a.m.
		Station B→Station C	1:46 a.m.–1:49 a.m.
		Station C→Station B	1:52 a.m.–1:55 a.m.
3	Retighten	Station B→Station C	2:06 a.m.–2:09 a.m.
		Station C→Station B	2:12 a.m.–2:15 a.m.
		Station B→Station C	2:18 a.m.–2:21 a.m.
		Station C→Station B	2:23 a.m.–2:26 a.m.

Note: (a) loosen fastener moments in monitoring areas #172, #164, and #160 were 1:22 a.m., 1:23 a.m. and 1:25 a.m., respectively. (b) retighten fastener moments in monitoring areas #160, #164, and #172 were 1:58 a.m., 2:00 a.m., and 2:02 a.m., respectively.

**Table 2:** The composition and size of the experimental dataset.

Label	Sample Source	Sample Size	
A: Fastener in the loose state	#160	4→32	96
	#164	4→32	
	#172	4→32	
B: Fastener in the normal state	#160	4 + 4 = 8	396
	#164	4 + 4 = 8	
	#172	4 + 4 = 8	
	#150~#180 (except for #160, #164, and #172)	31 × 12 = 372	

**Table 3:** The division and usage of the experimental dataset.

Dataset Division	Training Dataset Size		Test Dataset Size
	Pre-Training Stage	Fine-Tuning Stage	
Label A	67	67	29
Label B	277	67	119

**Table 4:** Performance evaluation of the CAE network from four indicators in the case of the test dataset.

Indicator	Accuracy	Precision	Recall	F1-Score
Result	0.8784	0.9902	0.8571	0.9189

**Table 5:** Performance comparison based on result analysis and discussion.

Item	Accuracy	Precision	Recall	F1-Score	Identifiability
Pseudo-Hilbert scan	0.8784	0.9902	0.8571	0.9189	87.39%
Raster scan	0.5743	0.8181	0.605	0.6957	chaos
Hilbert scan	0.7095	0.9043	0.7143	0.7589	75.63%

## Data Availability

The data presented in this study are available on request from the corresponding author.
